# Massive pulmonary embolism presenting initially as acute liver failure: A diagnostic challenge

**DOI:** 10.12669/pjms.42.(ICON26).15706

**Published:** 2026-04

**Authors:** Sara Soomro, Komal Baloch, Muneer Sadiq

**Affiliations:** 1 Sara Soomro, Department of Internal Medicine, Indus Hospital and Health Network, Karachi, Pakistan; 2 Komal Baloch, Department of Critical Care Medicine, Indus Hospital and Health Network, Karachi, Pakistan; 3 Muneer Sadiq, Department of Internal Medicine, Indus Hospital and Health Network, Karachi, Pakistan

**Keywords:** Acute, Massive, Liver Failure, Pulmonary Embolism, Rare

## Abstract

Pulmonary embolism is a common life-threatening emergency that can rarely be linked with acute liver dysfunction. Literature reports a complex link between deranged liver function tests and acute PE with or without severe valvular disease. Detailed literature review, till date, has reported only two case reports of patients presenting initially as acute liver failure and later diagnosed with acute pulmonary embolism. We report a case of a young female who was initially admitted with an impression of acute liver failure and was later diagnosed as having acute massive pulmonary embolism.

## INTRODUCTION

Pulmonary embolism (PE) is an acute, critical cardiovascular entity that ranks third among the most common types of cardiovascular diseases.^1^ While literature reports an incidence of PE to be 60 to 70 per 100,000, the actual figures may be much higher due to variable clinical presentations of PE.^2^ Even with advanced medical assessments, PE may be misdiagnosed, leading to a significant mortality of 30%.^2^

Acute liver failure (ALF) caused by PE is an extremely rare but critical medical complication that requires careful management. Literature reports a complex link between deranged liver function tests and acute PE with or without severe valvular disease.^3^ The primary mechanism of action may include PE leading to congestive heart failure (CHF), which results in hypoxic hepatitis. We report a case of a young female who was initially admitted with an impression of ALF and was later diagnosed as having acute massive PE.

## CASE REPORT

This is a case of 43 years old married female, recently diagnosed with hypertension and diabetes a few months back was on beta blockers, and non-complaint to medications. She had history of prolonged use of Homeopathic and herbal medications (almost 4-5 years) for her knee joint pains otherwise denied for other addictions or alcohol abuse, history of abdominal or pelvic surgery, trauma or history of immobility. Her history was also negative for autoimmune stigmata.

She presented with nausea, vomiting, and shortness of breath for three days without chest pain. On initial presentation in the Emergency Department (ED), she was an obese female who was oriented to time, place, and person but irritable (GCS-13-14/15) with tachycardia (109 beats per minute), hypotension (98/57mmHg), and tachypneic (32 breaths per minute), having saturation of 90% on room air with no fever. On detailed examination, she was icteric, with decreased air entry on bases of the chest, and her abdomen was soft, non-tender, with no visceromegaly. She had bilateral lower limb pitting oedema till mid-shins and had peripheral cyanosis in both feet. The rest of the examination was unremarkable.

She was immediately given intravenous fluids, and symptomatic management. Her initial venous blood gases were done, which showed metabolic acidosis with respiratory alkalosis (7.27/ PCO2:21/ HC03:12) and a lactate of seven. Her initial blood workup was sent, which reported a haemoglobin of 7.7mg/dl, serum creatinine of 2.93mg/dl and elevated AST of 1788 IU/L, ALT of 1500 IU/L and INR of 1.7 (Table-I). An ultrasound demonstrated hepatomegaly with fatty liver with patent vasculature. Considering her hypoxia and tachypnoea respiratory system was further evaluated with chest X-ray showed cardiomegaly but no other pulmonary pathology. Based on her irritability (hepatic encephalopathy), deranged INR and deranged creatinine, an impression of ALF secondary to viral hepatitis was made, and she was admitted to the High Dependency Unit. She was started on N-acetylcysteine (NAC) for her ALF. Viral hepatitis markers (Hep A/E/B/C) were reported as negative. Her Initial ECG only showed tachycardia, and echocardiography was planned however had to be postponed due to her irritability.

**Table-I:** Laboratory parameters of the patient throughout her illness course.

	1^st^ DAY	2^nd^ DAY	3^rd^ DAY
Hemoglobin (mg/dl)	7.7	8.2	9.7
White Blood Count (x10E9/L)	9.1	10.35	15.2
Platelets (x10E9/L)	223	160	164
Creatinine (mg/dl)	2.93	2.74	3.16
Total Bilirubin (mg/dl)	1.27	2.90	2.80
Direct Bilirubin (mg/dl)	0.65	1.99	2.13
GGT (U/L)	40	50	66
SGPT (U/L)	1788	1686	1783
Alkaline Phosphatase (U/L)	82	80	103
SGOT (U/L)	1533		
INR	1.71	1.91	2.03
Lactate	6	8	10
pH	7.265	7.270	7.372
PCO2	23.9	24.8	21.1
HCO3	12.6	13.4	14.8

**Fig.1:**
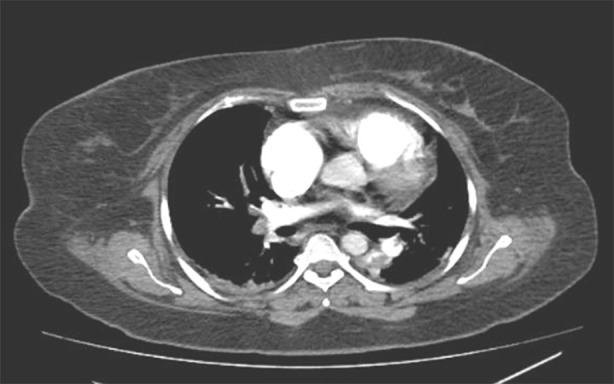
Computed tompography angiography showing pulmonary embolism in both pulmonary arteries.

But despite management, the patient’s clinical and laboratory parameters worsened within the next 48 hours (Table-I). She became drowsy, tachypnoeic, and had increased oxygen requirement with rising lactate. She was stepped up to the Intensive Care Unit and intubated electively on account of respiratory distress. She was transfused one pints of packed red blood cells. Her echocardiogram was commenced, which showed a dilated right atrium and ventricle with suspicion of McConnel’s sign. Ejection fraction was preserved without segmental wall abnormalities. Owing to this, a Computed Tomography (CT) Angiogram was ordered, which reported a large filling defect in the right main pulmonary artery and in the segmental and sub-segmental branches of left lower lobe arteries, representing pulmonary embolism (Fig.1). Liver was enlarged, with its tip extending into the left hypochondrium, appearing hypodense, representing fatty infiltration. An intrauterine copper device was in situ.

Since her INR was deranged and worsening, a risk vs benefit approach was taken with anticoagulation immediately planned with intravenous Streptokinase. However, before she could be started, she went into brady arrest and could not be revived even after a CPR of 30 minutes.

## DISCUSSION

An extensive literature review, till date, has reported only two case reports with patients presenting with ALF due to acute PE.^4^,^5^ All three patients, including ours, were young females who presented with non-specific symptoms of nausea, vomiting and shortness of breath. Detailed workup in all three patients was negative for viral and non-viral (drugs, toxins, autoimmune) causes of ALF except PE.

Aslan et al. reported that deranged liver function tests in acute PE patients were more profound in patients with severe hypoxemia or those with massive PE.^3^ This was true for all three patients, including ours, who had initially presented with massive acute PE. Inflammatory bowel disease (IBD) has been documented as a risk factor for venous thromboembolism.^6^ Rao et al. and Sengupta et al. patients were both diagnosed cases of Crohn’s disease that may have contributed to their presentation with acute PE.^4^,^5^ Our patient was a recently diagnosed case of diabetes and hypertension who had no risk factors for developing acute massive PE.

Echocardiography was the initial mode of investigation that led to the diagnosis of acute PE in all three patients. As per recent guidelines, bedside echocardiography is indicated for diagnosing suspected acute PE in high-risk patients.^7^ The presence of RV dysfunction, as assessed by echocardiography, can be an indication for thrombolysis, particularly in patients with hemodynamic instability.^7^,^8^ Our patient’s echocardiography had right ventricular free wall akinesis with sparing of the apex, called as Mcconnel’s sign. As per some studies, McConnel’s sign is highly specific for acute PE.^9^

In all three patients, diagnosis of acute PE was delayed for 48-72 hours till after admission, hence a delay in therapeutic anticoagulation. Bharat et al.’s and Sengupta et al’s patients were immediately started on intravenous heparin after diagnosis was confirmed by CT Angiography with good outcomes.^4^,^5^ Our patient was planned for intravenous streptokinase; however, she developed sudden cardiac arrest before she could be thrombolysed and died.

## CONCLUSION

Given the complexities, clinicians must maintain a high index of suspicion for pulmonary embolism in patients presenting initially as acute liver failure. Early imaging and a multidisciplinary approach are crucial for timely diagnosis and management to improve patient outcomes.
